# Supplementary material for: Impact of the COVID-19 Pandemic on Incidence and Observed Survival of Malignant Brain Tumors in Belgium
Source: Cancers (Basel). 2023 Dec 21;16(1):63. doi: 10.3390/cancers16010063 (PMC10778220; doi:10.3390/cancers16010063)
Supplement: Supplementary file 1 [file cancers-16-00063-s001.zip › cancers-2734147-supplementary.pdf]

Table S1. Included ICD-O-3 codes for data extraction from BCR database

Malignant brain tumor subtype	Topography	ICD-O-3 morphology codes	Behavior
All malignant brain tumors	C71	8000*;8070;8140;8158;8272;8720;8728;8680;8682;8693;8800-8802; 8805-8806;8810;8815;8830;8850;8890;8900;8910;9060; 9064-9065; 9070-9071;9080;9084-9085;9100;9120;9133;9140; 9150; 9180;9220;9260;9362;9364;9380-9382;9385;9390-9393; 9395-9396;9400-9401;9410-9411;9420;9421;9424-9425;9430; 9440-9442;9445;9450-9451;9470-9478;9490;9501;9505;9508; 9530;9538-9540;9560-9561;9571;9590-9591;9596;9650;9663; 9670-9671;9673;9680;9684;9687-9688;9690-9691;9698-9699; 9702;9712;9714-9715;9728-9729;9731;9734-9735;9749-9751; 9754-9755;9766;9811-9812;9823;9826;9836-9837;9861;9930	3
Glioblastoma	C71	9440-9442;9445	3
Hematolymphoid tumors	C71	9590-9591;9596;9650;9663;9670-9671;9673;9680;9684;9687-9688;9690-9691;9698-9699;9702;9712;9714-9715;9728-9729; 9731;9734-9735;9749-9751;9754-9755;9766;9811-9812;9823; 9826;9836-9837;9861;9930	3
Other malignant brain tumors	C71	8000*;8070;8140;8158;8272;8720;8728;8680;8682;8693;8800-8802;8805-8806;8810;8815;8830;8850;8890;8900;8910;9060; 9064-9065;9070-9071;9080;9084-9085;9100;9120;9133;9140; 9150;9180;9220;9260;9362;9364;9380-9382;9385;9390-9393; 9395-9396;9400-9401;9410-9411;9420;9421;9424-9425;9430; 9450-9451;9470-9478;9490;9501;9505;9508;9530;9538-9540; 9560-9561;9571	3

*Defined as separate subgroup (unspecified tumors) in Fig. 4b
